# High rate of complications and radiographic loosening of the biaxial total wrist arthroplasty in rheumatoid arthritis: 32 wrists followed for 6 (5–8) years

**DOI:** 10.3109/17453674.2011.636669

**Published:** 2011-11-25

**Authors:** Djoeke van Harlingen, Petra JC Heesterbeek, MaartenJde Vos

**Affiliations:** ^1^Department of Orthopaedics; ^2^Department of Research, Development and Education, Sint Maartenskliniek, Nijmegen; ^3^Department of Orthopaedics, Tergooiziekenhuizen, Hilversum, the Netherlands

## Abstract

**Background and purpose:**

The third generation of total wrist arthroplasty (TWA) was designed to solve the early loosening problem, but there have been few long-term follow-ups. We present the outcome of the biaxial total wrist prosthesis (no longer available) after 5–8 years of follow-up.

**Patients and methods:**

40 biaxial wrist prostheses were implanted uncemented in 36 patients with rheumatoid arthritis. 32 wrists were followed clinically and radiographically. 7 prostheses had been revised at median 21 (8–71) months; 1 patient died from an unrelated cause. Mean follow-up of the remaining 32 wrists was 6 (5–8) years. Kaplan-Meier survival analysis was performed with revision defined as failure.

**Results:**

Survival after 7 years was 81% (95% CI: 64–91). There were 31 complications. 22 wrists showed radiographic loosening. Range of motion improved, except for pronation. The mean DASH score improved and the median postoperative pain score (from 0 to 10) was 0 (0–6) at rest and 0 (0–7) during activity.

**Interpretation:**

One quarter of the prostheses had been revised and radiographic loosening had occurred in two thirds of the cases. Radiographic and clinical follow-up is therefore necessary for patients with this implant.

The wrist is affected in one half of patients with rheumatoid arthritis during the first 2 years after onset of the disease, increasing to > 90% after 10 years (Trieb 2008). The first generation of wrist implants was developed in 1967 (Swanson 1973). It consisted of one flexible piece of silicone hinge. The initial results were promising, but stress at the silicone implants caused disintegration of the silicone with severe inflammatory reactions and mechanical failure. The second-generation implants were developed in the early 1970s (Volz 1976, Meuli 1980). They usually included 2 metal components that articulated by means of a ball-and-socket or hemispheric design. The main problem with these prostheses was loosening of the metacarpal component, joint imbalance, and dislocation. The third generation, such as the biaxial wrist prosthesis, was designed to solve these problems (Costi et al. 1998). Many prostheses of this type have been used in the past, but no long-term evaluation has been published.

Previous literature on the biaxial wrist prosthesis showed satisfactory pain relief, an improvement in range of motion, and satisfactory function after follow-up of 6, 3, and 4 years but also loosening, infections, and dislocation (Cobb and Beckenbaugh 1996, Courtman et al. 1999, Takwale et al. 2002). We analyzed the outcome of this prosthesis in 36 patients (40 prostheses) after 6 (5–8) years of follow-up.

## Patients and methods

### Patients

**Figure 1.**
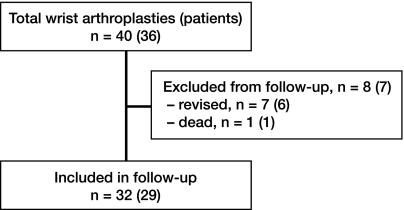
Flow chart of follow-up.

We included 36 adult patients (28 women) who received a primary biaxial total wrist arthroplasty (bilateral in 4 patients) for rheumatoid arthritis between December 2000 and September 2004. Median age at the time of surgery was 54 (18–74) years. The primary indication for surgery was severe wrist pain accompanied by wrist deformity and/or limitation of motion and function. At the follow-up, 32 total wrist arthroplasties could be evaluated, 7 prostheses had been revised with implant removal, and 1 patient had died from an unrelated cause (Figure 1). The mean follow-up of the remaining implants was 6 (5–8) years.

### Operation

In all patients, a biaxial total wrist arthroplasty designed by Beckenbaugh was used (DePuy Orthopaedics Inc, Leeds, UK; no longer available: the producer withdrew the implant from the market because it was no longer profitable). It has a rounded, unconstrained, articulating interface, oriented in the plane of wrist movement. The prosthesis is composed of a metacarpal (distal) and a radial (proximal) component and the stems have porous-coated surfaces. The distal component consists of a larger stem for insertion into the third metacarpal and a small stud for insertion into the trapezoid to stabilize it during rotation.

Prophylactic antibiotic (1 g cefazoline) was given intravenously 20–30 min before the start of surgery. A tourniquet was applied to the upper arm. The surgical technique was according to Cobb and Beckenbaugh (1996). Neither component was cemented. Postoperative care consisted of 7–10 days of splint immobilization followed by a supervised program of physiotherapy. A removable splint was used for 4 weeks and was only taken off when the exercises were performed. A night splint was used for 3 months. All operations were performed by or directly supervised by the same orthopedic surgeon (MV).

### Radiographic measurements

**Figure 2.**
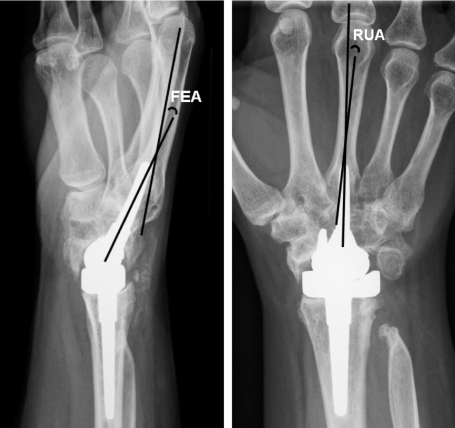
Left panel: The flexion-extension angle (FEA) is the angle between the line through the middle of the metacarpal stem and the middle of the third metacarpal on the lateral radiograph. Right panel: Radial-ulnar angle (RUA) is the angle between the middle of the metacarpal stem and the middle of the third metacarpal on the anterior-posterior radiograph.

Postoperative radiographs at follow-up (anterior-posterior and lateral) were evaluated for radiolucent lines around the implants, subsidence and migration of the metacarpal implant, and erosion of the tip of the implant through the cortex of the third metacarpal. To measure the radiolucent lines, we used radiographic zones according to Cobb and Beckenbaugh (1996). The metacarpal component consists of 6 zones and the radial component of 5. The width of the radiolucent line was measured in mm on the follow-up radiograph (anterior-posterior view) for each different zone. A radiolucent line of 2 mm or more was defined as radiographic loosening. To determine subsidence of the metacarpal component, we measured the length from the tip of the metacarpal stem to the distal end of the third metacarpal on the primary postoperative radiograph (anterior-posterior view) and on follow-up radiographs. Subsidence was calculated by subtracting the two measurements. Angular migration was assessed on the primary postoperative radiograph, and at follow-up by measuring flexion-extension tilt (FEA on the lateral view) and by radio-ulnar angulation (RUA on the anterior-posterior view) (Figure 2). There may be measurement error due to variation in projection angle when the radiograph is taken. According to Cobb and Beckenbaugh (1996), this may be around 3 mm. We therefore decided that 5 mm or more was significant for subsidence, and migration of 10 degrees or more for the flexion-extension tilt and the radio-ulnar angulation. Radiographic loosening was defined as a radiolucent line equal to or greater than 2 mm in at least 1 zone, erosion of the tip of the implant through the cortex, subsidence equal to or greater than 5 mm, or migration equal to or greater than 10 degrees.

To examine the reproducibility of the radiographic measurements, we assessed the inter- and intra-observer variability. 12 randomly chosen radiographs were independently measured for subsidence and angular migration by 2 observers. One observer, blinded regarding the previous measurements results, measured all the radiographs a second time. The second observer measured the radiographs once. The inter-observer variability was calculated between the first measurements of observer 1 and the measurements of observer 2 using the method of Bland and Altman (1986). Intra-observer variability was calculated between the 2 measurements of observer 1. The inter- and intra-observer variability for the distance of the third metacarpal were acceptable (Table 1). Because the 95% prediction limits for the radiographic measurements were within 5 mm and 10 degrees, we used these measurements to determine which prostheses had subsidence and/or angular migration (Table 1).

**Table 1.** 95% prediction limits of radiographic measurements. To assess the variability between 2 measurements (either intra- or inter-observer), the difference between these 2 measurements was plotted against their mean. The mean difference would be expected to be zero since the same method was used. With the help of the mean difference and the standard deviation of the differences, the limits of agreement, also known as the 95% prediction limits, were calculated (Bland and Altman 1986)

	Intra-observer	Inter-observer
Third metacarpal distance (mm)	3.1	3.4
Flexion-extension tilt (degrees)	6.5	4.9
Radio-ulnar angulation (degrees)	3.8	4.6

### Clinical evaluation

Preoperative data that had been collected included range of motion (extension, flexion, radial deviation, ulnar deviation, pronation, and supination) measured by goniometry and DASH score in the validated Dutch translation (Disability of the Arm, Shoulder, and Hand questionnaire) (Veehof et al. 2002, www.dash.iwh.on.ca). A score of 0 reflected no disability and the minimal clinically important difference was chosen as being a change of 12.6 points or more (Schmitt and Di Fabio 2004). Severity of destruction of the wrist was evaluated by the Larsen score on the anterior-posterior radiograph (Larsen et al. 1977).

29 patients (32 prostheses) were available for follow-up evaluation; they were examined by an independent investigator (DvH). To assess the function of the prostheses, an objective grip strength and range of motion test was performed together with a subjective DASH- and pain score. The wrists were assessed for pain by scoring on an ordinal scale from 0 to 10, at rest and during activity. Range of motion was measured with a goniometer according to the same protocol as that used preoperatively. To determine the remaining amount of strength of the hand in kg, we calculated the average value after 3 attempts using a Baseline Hydraulic Hand Dynamometer (Fabrication Enterprises, Inc., New York, NY). The Baseline Hydraulic Pinch Gauge (Fabrication Enterprises) was used to measure 3 different types of strength; each is given as an average of 3 attempts. Key pinch strength was measured between thumb pad and the radial side of the second digit, tripod grip strength between thumb pad and the palmar surfaces of the second and third digits, and tip pinch strength between the tip of the thumb and the tip of the second digit. Also, patient satisfaction was scored on a 5-point scale: very satisfied, satisfied, moderate, or unsatisfied. Complications were noted and classified as intraoperative or postoperative complications.

### Statistics

Kaplan-Meier survival analysis was used to estimate the cumulative probability of remaining free of revision. The differences between preoperative and postoperative range of motion and DASH score were analyzed using a non-parametric Wilcoxon signed-rank test for data that were not normally distributed (pronation and supination) and the parametric Student t-test was used for normally distributed data (flexion, extension, radial deviation, ulnar deviation, and DASH). Significance was set at a p-value of less than 0.05.

## Results

### Complications

There were 4 intraoperative and 27 postoperative complications, in 22 prostheses (21 patients) (Table 2). Difficulties in closure of the capsule were solved with the use of a Gore-Tex Soft Tissue Patch (WL Gore and Associates Inc., Flagstaff, AZ). Breakout of the stem through the cortex of third metacarpal was noticed on the primary postoperative radiographs in 2 patients who were treated with 14 days of splint immobilization. One of these patients developed a limited range of motion.

**Table 2.** Complications. The numbers in parentheses indicate the number of prostheses

Complication	Solution	Further complications	Need for revision
Intraoperative (4)			
Severe soft bone tissue (1)	Prostheses cemented		
Difficulty in wound closure (1)	Soft tissue patch		
Breakout distal component (2)	14 days splint immobilization	Limited ROM (1) **^a^**	
Postoperative (27)			
Dislocations (7)			
Direct postoperative (2)	14 days of immobilization		
Late (4)	Reposition, expectative		
Ulnar instability (1)	Flexor carpi ulnar transposition		
Infections (3)			
Superficial (1)	Antibiotics (1)	Limited ROM + loosening (1)	
Deep (2)	Removal of prosthesis (2)		Deep infections (2)
Limited range of motion (7)	Operative release of capsule (3)		
	Manipulation under anesthetic (4)		
Nerve damage (3)			
CTS (2)	CTS release (2)		
Sensible disorder ulnar nerve (1)	Expectative (1)		
Ulnar pain (1)	Further resection ulna		
Wound dehiscence (1)	Operative debridement		
Need for revision (5)	Removal prosthesis (5)	Not available	
Loosening (3)			Loosening (3)
Malpositioning (1)			Malpositioning (1)
Breakout of distal component (1)			Breakout of distal component (1)

**^a^**One patient had a superficial infection, limited range of motion, and loosening (in order of appearance). This loosening is one of the three mentioned under “Need for revision”.

The patient with the ulnar instability received a soft tissue reconstruction according to Tsai and Stilwell (1984), and the other 6 wrists were successfully treated nonoperatively. Of the 3 infections, the one that was superficial developed a limited range of motion. The same prosthesis had symptoms of loosening 5 years later. The other 2 infections resulted in implant removal. The 6 prostheses with limited range of motion all had an improvement after manipulation or operative release of the capsule. Due to permanent pain on the ulnar side with a dissociation of the distal-radial-ulnar joint in one patient, we performed a further resection of the ulna, which relieved the pain. 7 wrists were revised with removal of the prosthesis (Table 3).

**Table 3.** Revisions

Case	Months in situ	Reason for revision	Age at operation	Larsen pre-operatively	Dominant side (–/+)
1	22	Loosening	39	I	–
2	9	Malpositioning	23	III	–
3	66	Loosening	63	I	+
4	18	Infection	66	V	+
5 **^a^**	71	Breakout of distal component	63	II	–
6 **^a^**	8	Infection	64	IV	+
7	59	Loosening	61	III	+

**^a^**5 and 6 are the same patient with bilateral total wrist arthroplasty.

### Radiographic evaluation

**Figure 3.**
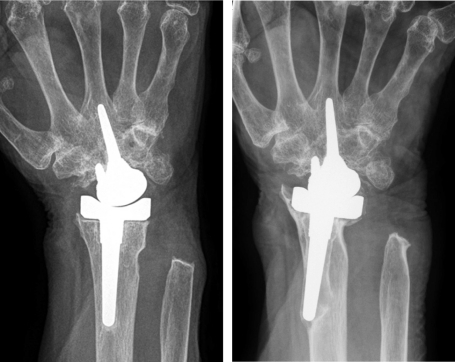
Left: 1 year postoperatively. Right: 5.5 years postoperatively showing severe loss of bone stock with migration, collapse, and breakthrough of the cortex of the radial component.

Preoperatively, 2 patients were Larsen stage V, 20 were stage IV, 7 were stage III, 7 were stage II, and 4 patients were stage I. At follow-up, 22 prostheses had radiographic loosening. 5 had a breakout of the third metacarpal cortex (2 intraoperatively), 15 had subsidence of ≥ 5 mm, 7 had angular migration of ≥ 10 degrees, and 12 had a minimum of 1 radiolucent line ≥ 2 mm in at least 1 zone. Some patients had more than 1 radiographic category of loosening (Figure 3).

### Clinical evaluation

*Range of motion*. The range of motion improved statistically significantly after surgery, except for pronation (Table 4).

**Table 4.** Range of motion and DASH score in 32 wrists

Motion (°)	Preoperatively	Postoperatively	p-value	n
Extension **^a^**	18 (17)	28 (17)	0.004	30
Flexion **^a^**	21 (14)	29 (17)	0.04	30
Radial deviation **^a^**	5 (6)	10 (8)	0.03	22
Ulnar deviation **^a^**	4 (6)	19 (13)	< 0.001	22
Pronation **^b^**	80 (5–90)	85 (0–95)	0.3	17
Supination **^b^**	70 (0–90)	90 (35–110)	0.02	17
DASH (0–100) **^a^**	66 (25)	34 (24)	< 0.001	31

**^a^**Values are mean (SD)

**^b^** Values are median (range) Because of some missing preoperative data, statistical analysis was only performed with the data of patients having matching pre- and postoperative data. Student's t-test was used for normally distributed data, and the Wilcoxon signed-rank test was used as a non-parametric alternative.

*DASH.* The DASH score at follow-up improved by 48% (p < 0.001). A preoperative DASH score was missing in 1 patient (n = 31). 22 wrists had an improvement in DASH score of more than 12.6 points, and 1 patient had a deterioration of 55 points.

*Pain.* All patients reported a decrease in pain at the time of follow-up. The median postoperative pain score was 0 (range 0–6) at rest and 0 (range 0–7) during activity.

*Patient satisfaction.* 18 patients were very satisfied with the result of their prosthesis, 9 were satisfied, 4 were moderately satisfied, and 1 patient was not satisfied. The reasons reported by the moderately satisfied patients were limited range of motion (3 cases) and limited range of motion with pain (1 case). The patient who was not satisfied gave repeated dislocations of the prosthesis as the cause.

*Strength.* There were high inter-individual variations in strength. The mean grip strength at follow-up was 13 (SD 8) kg. Tip pinch was 2.8 (SD 1.2) kg, tripod grip strength 4.5 (SD 2.3) kg, and key pinch was 5.7 (SD 2.7) kg. In many patients, other joints on the ipsilateral side of the prosthesis were also affected; 17 patients had dysfunction of the hand, due to deformities and/or pain. 5 patients also had complaints of the elbow and 8 of the shoulder.

### Survival

**Figure 4.**
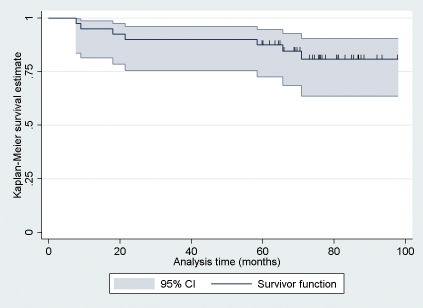
Cumulative survival of 40 prostheses with revision surgery defined as failure event. The small vertical spikes represent the censored data.

The mean cumulative survival after 87 months was 81% (95% CI: 64–91) (Figure 4). There were 4 relatively early revisions (range 8–22 months) and 3 later revisions (range 59–71 months). After evaluating the follow-up radiographs, 2 additional wrists were scheduled for revision. These 2 patients were not included as revisions in the survival analysis.

## Discussion

The biaxial wrist implant, a third-generation wrist prosthesis, was developed to overcome problems with joint imbalance, dislocations, and loosening of the metacarpal component common in the earlier prostheses. In our study of this implant, most patients were satisfied but we found numerous complications with a mean cumulative survival of 81% at 7 years. In addition to revision, there was radiographic evidence of loosening in 22 of the 32 wrists. The implant survival was similar to that in previous reports, but the radiographic evidence of loosening was higher (Cobb and Beckenbaugh 1996, Takwale et al. 2002).

Cobb and Beckenbaugh (1996) followed 57 prostheses for a minimum of 5 years. Radiographic loosening occurred in 14 cases. The survival, with revision as endpoint, at 5 years was 83%. The largest study was performed by Takwale et al (2002), with a mean follow-up of 4 years. They reported a probability of survival for the biaxial total wrist replacement of 83% at 8 years with revision surgery as endpoint. We found a high proportion of radiographic loosening. Most of the patients were asymptomatic at that time, which has also been found in other studies (Torchia et al. 1997, Martin et al. 2005, van der Lugt et al. 2010). When a prosthesis has gradually loosened, there will be progressive loss of bone stock (Talwalkar et al. 2005). It is therefore necessary that patients with a biaxial total wrist prosthesis be closely followed, to recognize radiographic loosening while sufficient bone stock remains for revision—even if they are asymptomatic.

We found 31 complications in 21 patients. Cobb and Beckenbaugh (1996) had a similar percentage of complications whereas Takwale et al. (2002) had fewer complications. Other authors have reported some complications; the definitions may not have been the same, however, and for this reason comparison of complication rates is not reliable (Lirette and Kinnard 1995, Courtman et al. 1999, Stegeman et al. 2005). A fourth generation of prostheses is now being used. They are designed to solve the problems of dislocation and loosening, by preserving the distal radio-ulnar joint. However, no long-term evaluations have been published yet.

Despite the high rate of complications, a surprisingly large proportion of patients (27/32), were “very satisfied” or “satisfied” with the result of the operation. Functional range of wrist motion in healthy subjects was determined by Palmer et al. (1985) at 5˚ of flexion, 30˚ of extension, 10˚ of radial deviation, and 15˚ of ulnar deviation. Ryu et al. (1991) reported that 40˚ of wrist extension, 40˚ of wrist flexion, and a total of 40˚ of radial-ulnar deviation is needed to perform most activities of daily living. Mean range of motion in the present study was 29˚ of flexion, 28˚ of extension, 10˚ of radial deviation, and 19˚ of ulnar deviation. Thus, functional range of motion can largely be achieved. The postoperative range of motion was comparable to those in previous studies (Lirette and Kinnard 1995, Cobb and Beckenbaugh 1996, Takwale et al. 2002).

22 of 31 cases had an improved postoperative DASH score (more than 12.6 points); we may therefore conclude that patients gained functional benefit from the surgery. The remaining strength after surgery was comparable with that descibed in the literature for rheumatoid arthritis (Bodur et al. 2006). Wrist prostheses have no negative effect on strength of the hand and fingers for patients with rheumatoid arthritis.

We had no preoperative pain scores; thus, no comparison with the postoperative score was possible. Considering that the primary indication for surgery was severe wrist pain, we remain confident that the patients had reduced pain scores.We terminated the survival analysis when the number of patients remaining in the sample reached 10% of the initial population. For our study, this was at 87 months when only 4 patients remained. However, because of the small sample size and some early failures, the confidence interval bands were broad quite early in the analysis. Another issue is inclusion of bilateral cases in the statistical analysis. 4 patients had bilateral total wrist prostheses and 1 patient had both implants revised. We performed a second survival analysis including only the first operated wrist of the bilateral cases, and the survival rate was similar with only a very slightly broader confidence interval.

In conclusion, the biaxial total wrist prosthesis in patients with rheumatoid arthritis had a mean cumulative survival for revision after 7 years of 81% (95% CI: 64–91) with good pain relief, with improved motion, and with most patients satisfied. However, the high complication rate and a worrying amount of radiographic loosening after 5 years are problematic. Regular clinical and radiographic follow-up is therefore necessary.
